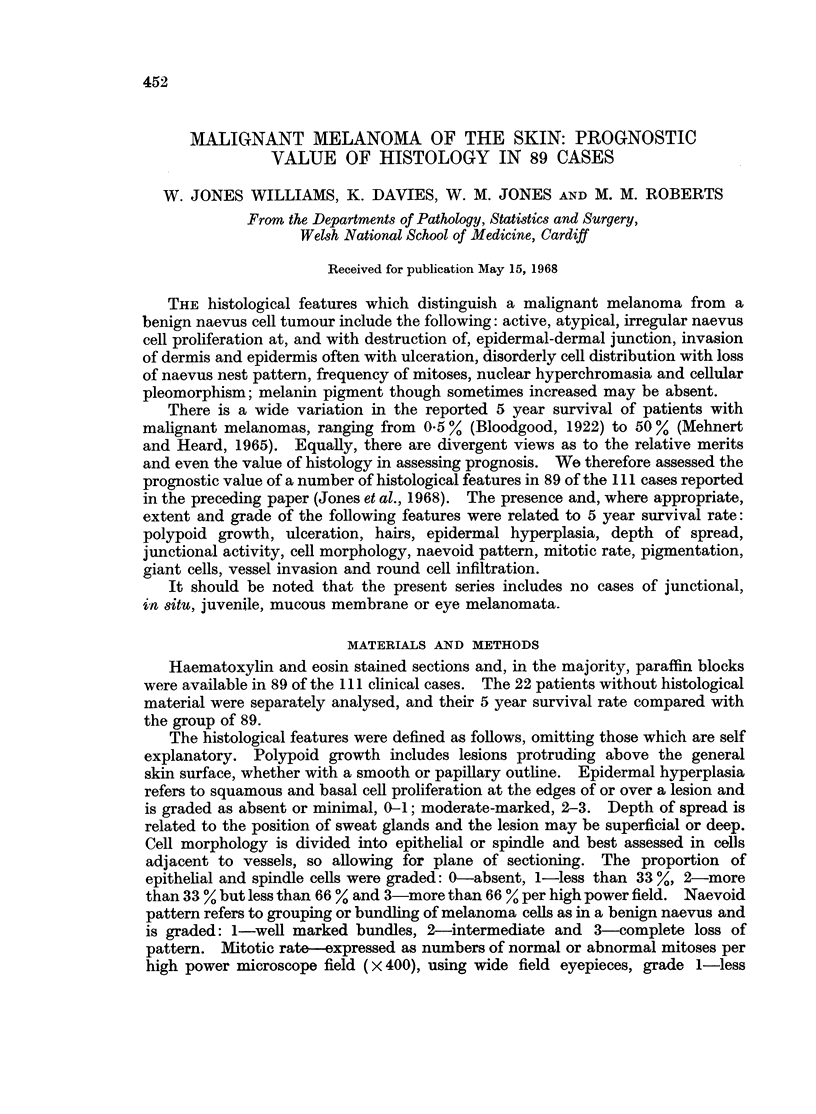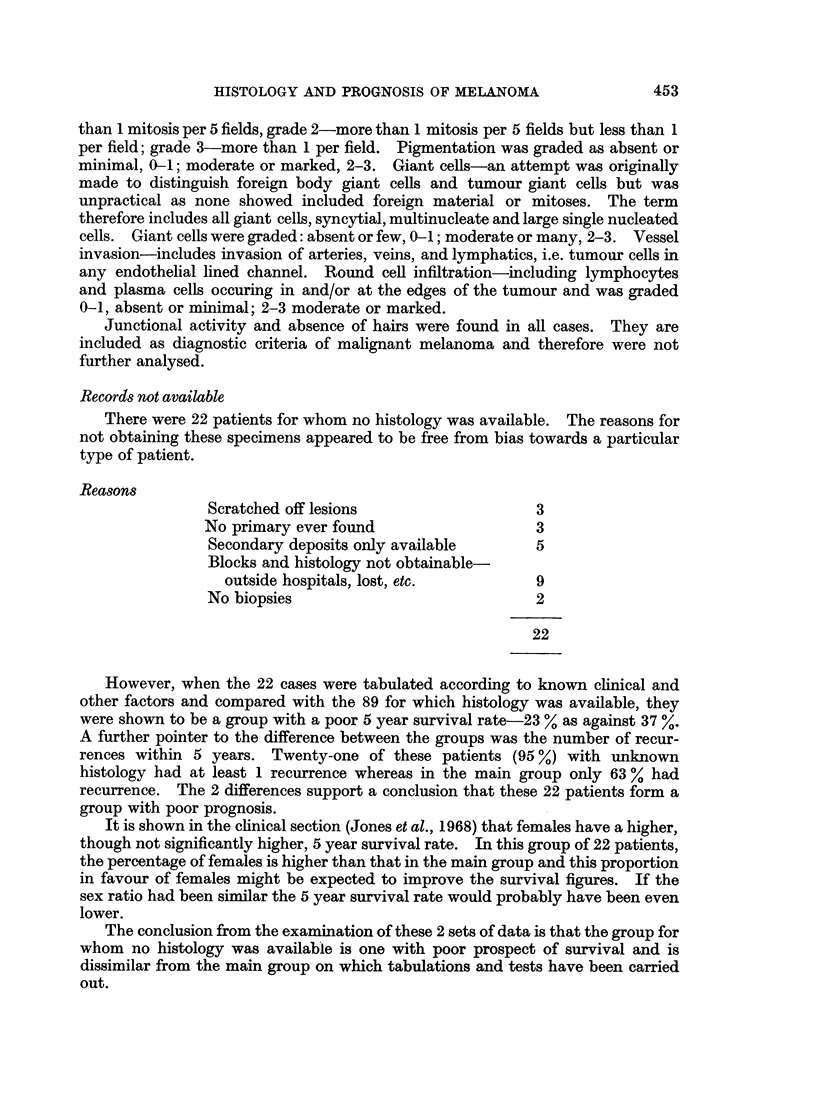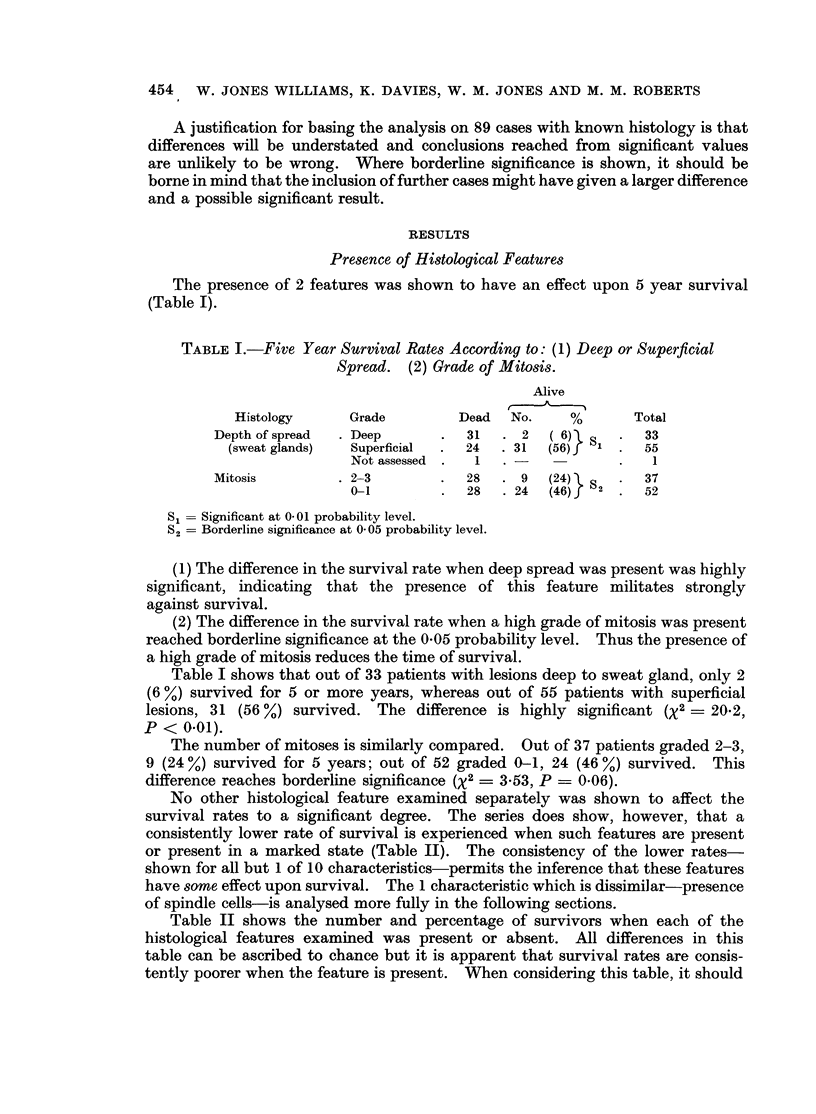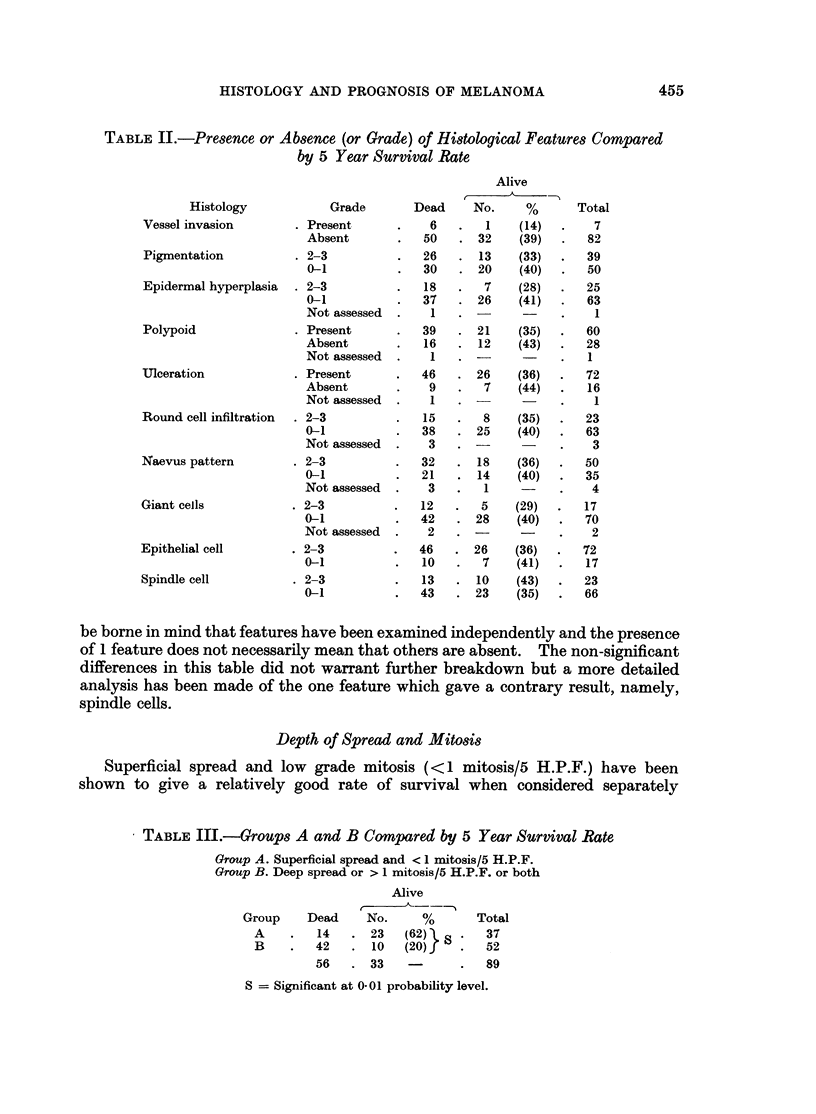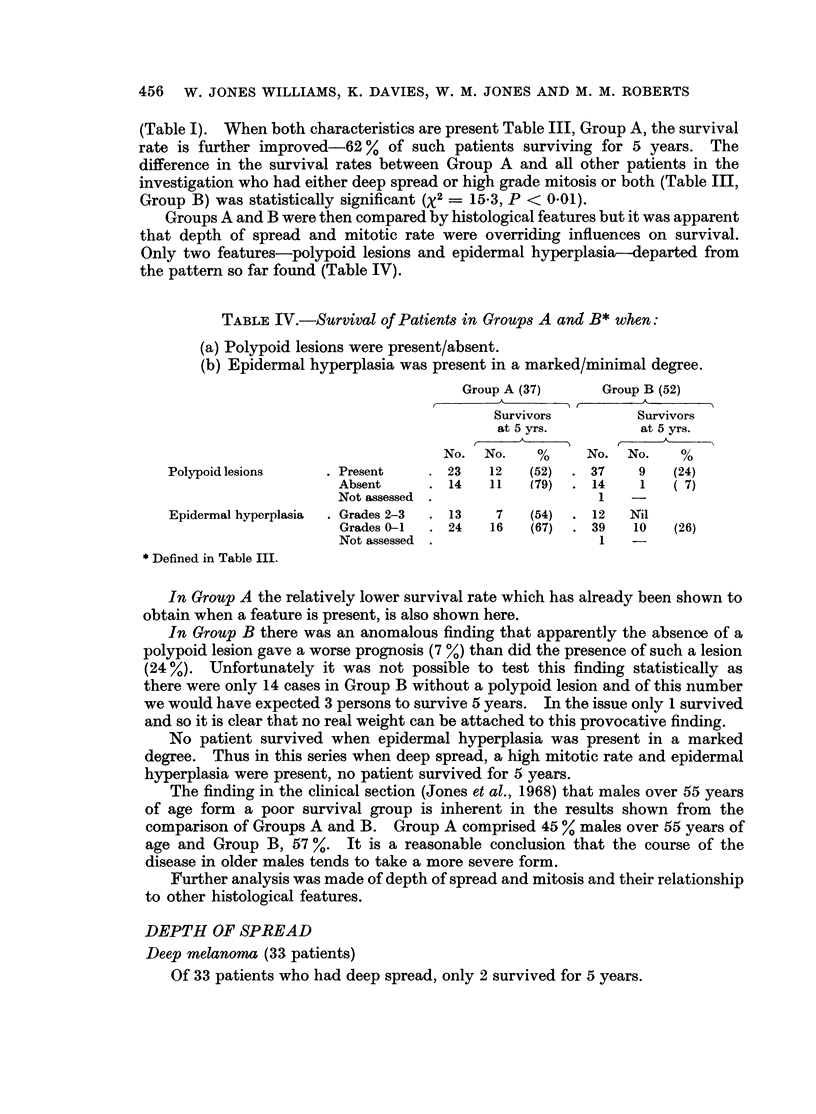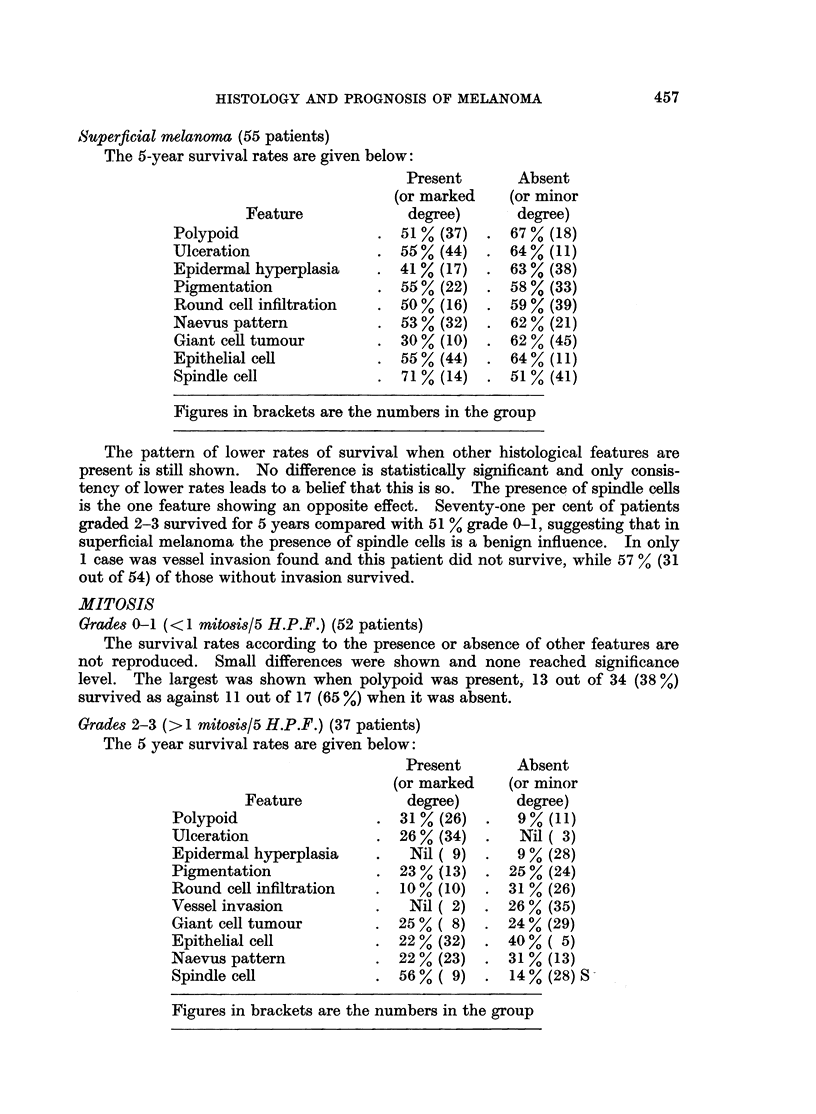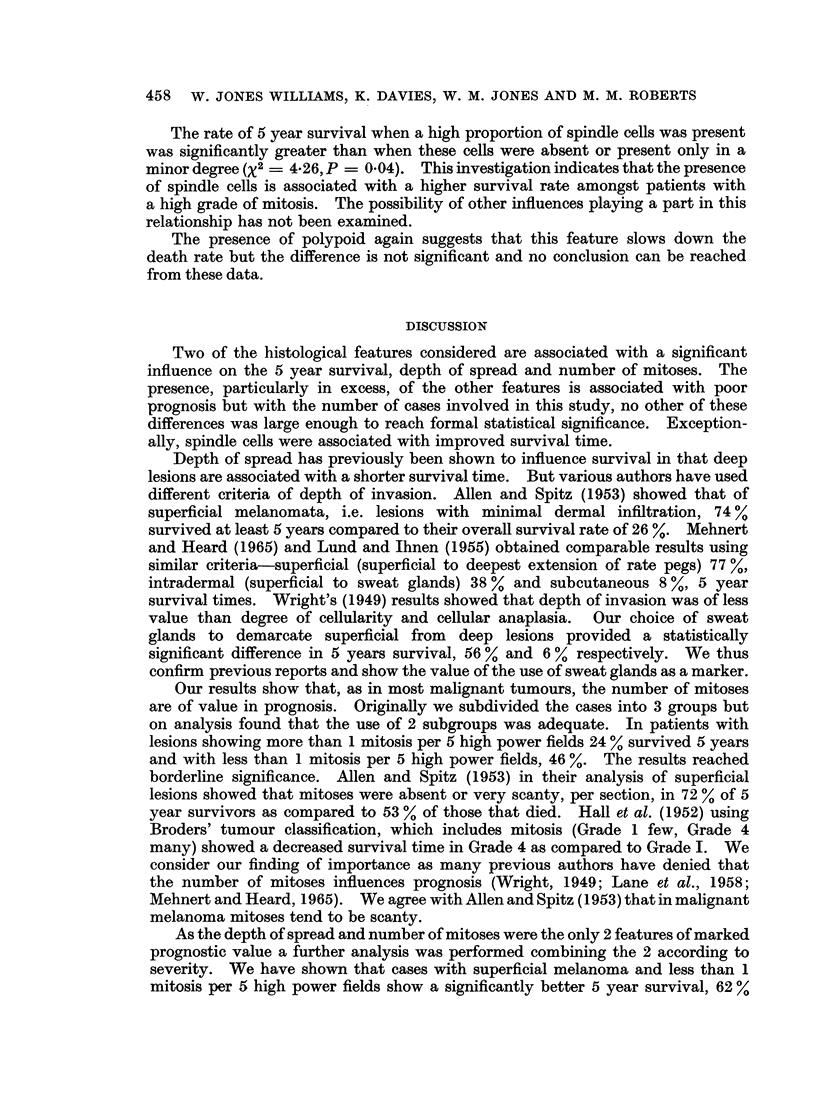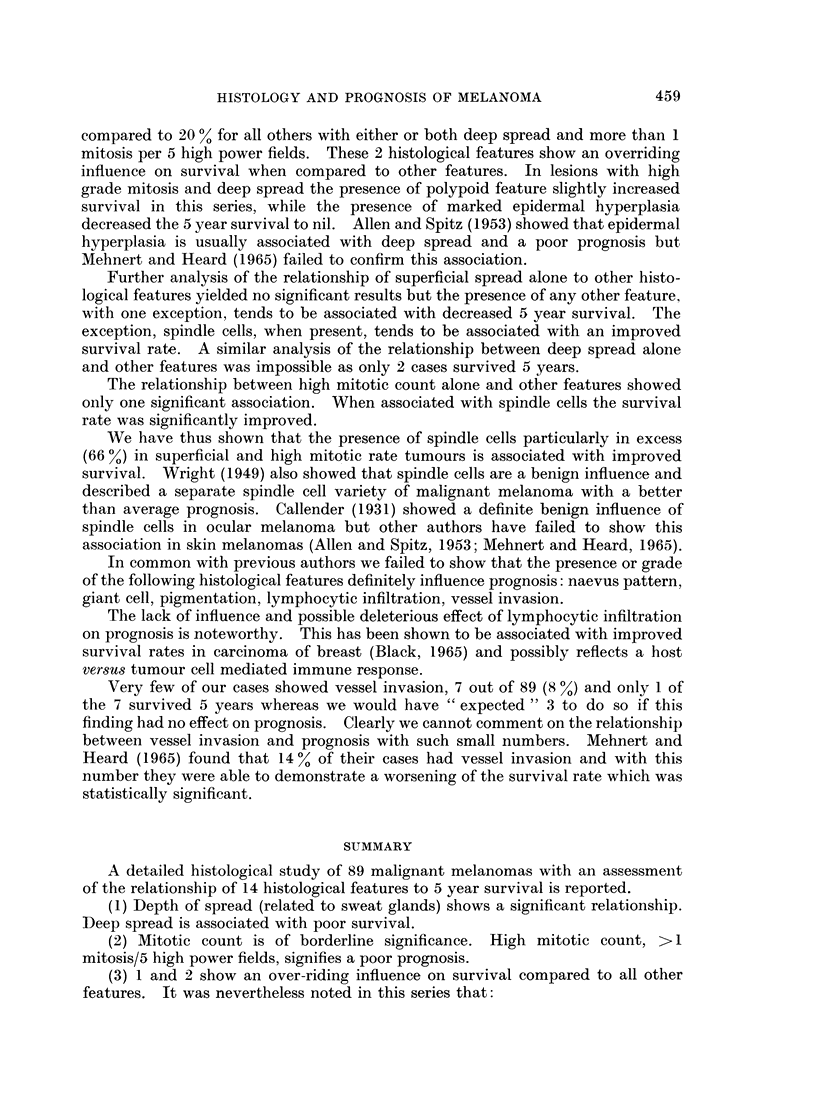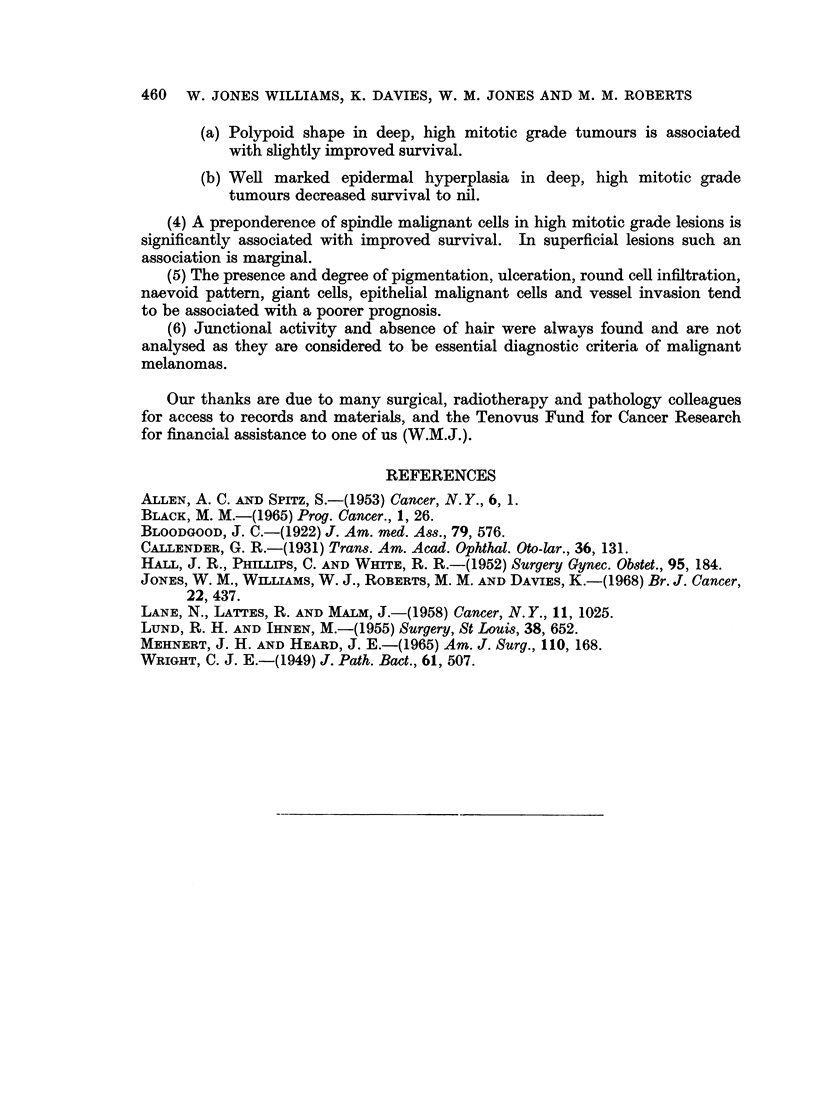# Malignant melanoma of the skin: prognostic value of histology in 89 cases.

**DOI:** 10.1038/bjc.1968.52

**Published:** 1968-09

**Authors:** W. J. Williams, K. Davies, W. M. Jones, M. M. Roberts


### MALIGNANT MELANOMA OF THE SKIN: PROGNOSTIC VALUE OF HISTOLOGY IN 89 CASES

W. JONES WILLIAMS, K. DAVIES, W. M. JONES AND M. M. ROBERTS

*From the Departments of Pathology, Statistics and Surgery, Welsh National School of Medicine, Cardiff*

Received for publication May 15, 1968

THE histological features which distinguish a malignant melanoma from a benign naevus cell tumour include the following: active, atypical, irregular naevus cell proliferation at, and with destruction of, epidermal-dermal junction, invasion of dermis and epidermis often with ulceration, disorderly cell distribution with loss of naevus nest pattern, frequency of mitoses, nuclear hyperchromasia and cellular pleomorphism; melanin pigment though sometimes increased may be absent.

There is a wide variation in the reported 5 year survival of patients with malignant melanomas, ranging from 0·5 % (Bloodgood, 1922) to 50 % (Mehnert and Heard, 1965). Equally, there are divergent views as to the relative merits and even the value of histology in assessing prognosis. We therefore assessed the prognostic value of a number of histological features in 89 of the 111 cases reported in the preceding paper (Jones *et al.*, 1968). The presence and, where appropriate, extent and grade of the following features were related to 5 year survival rate: polypoid growth, ulceration, hairs, epidermal hyperplasia, depth of spread, junctional activity, cell morphology, naevoid pattern, mitotic rate, pigmentation, giant cells, vessel invasion and round cell infiltration.

It should be noted that the present series includes no cases of junctional, *in situ*, juvenile, mucous membrane or eye melanomata.

### MATERIALS AND METHODS

Haematoxylin and eosin stained sections and, in the majority, paraffin blocks were available in 89 of the 111 clinical cases. The 22 patients without histological material were separately analysed, and their 5 year survival rate compared with the group of 89.

The histological features were defined as follows, omitting those which are self explanatory. Polypoid growth includes lesions protruding above the general skin surface, whether with a smooth or papillary outline. Epidermal hyperplasia refers to squamous and basal cell proliferation at the edges of or over a lesion and is graded as absent or minimal, 0–1; moderate-marked, 2–3. Depth of spread is related to the position of sweat glands and the lesion may be superficial or deep. Cell morphology is divided into epithelial or spindle and best assessed in cells adjacent to vessels, so allowing for plane of sectioning. The proportion of epithelial and spindle cells were graded: 0—absent, 1—less than 33 %, 2—more than 33 % but less than 66 % and 3—more than 66 % per high power field. Naevoid pattern refers to grouping or bundling of melanoma cells as in a benign naevus and is graded: 1—well marked bundles, 2—intermediate and 3—complete loss of pattern. Mitotic rate—expressed as numbers of normal or abnormal mitoses per high power microscope field (×400), using wide field eyepieces, grade 1—less than 1 mitosis per 5 fields, grade 2—more than 1 mitosis per 5 fields but less than 1 per field; grade 3—more than 1 per field. Pigmentation was graded as absent or minimal, 0–1; moderate or marked, 2–3. Giant cells—an attempt was originally made to distinguish foreign body giant cells and tumour giant cells but was unpractical as none showed included foreign material or mitoses. The term therefore includes all giant cells, syncytial, multinucleate and large single nucleated cells. Giant cells were graded: absent or few, 0–1; moderate or many, 2–3. Vessel invasion—includes invasion of arteries, veins, and lymphatics, i.e. tumour cells in any endothelial lined channel. Round cell infiltration—including lymphocytes and plasma cells occuring in and/or at the edges of the tumour and was graded 0–1, absent or minimal; 2–3 moderate or marked.

Junctional activity and absence of hairs were found in all cases. They are included as diagnostic criteria of malignant melanoma and therefore were not further analysed.

*Records not available*

There were 22 patients for whom no histology was available. The reasons for not obtaining these specimens appeared to be free from bias towards a particular type of patient.

*Reasons*

| | |
|---|---|
| Scratched off lesions | 3 |
| No primary ever found | 3 |
| Secondary deposits only available | 5 |
| Blocks and histology not obtainable—outside hospitals, lost, *etc.* | 9 |
| No biopsies | 2 |
| | 22 |

However, when the 22 cases were tabulated according to known clinical and other factors and compared with the 89 for which histology was available, they were shown to be a group with a poor 5 year survival rate—23 % as against 37 %. A further pointer to the difference between the groups was the number of recurrences within 5 years. Twenty-one of these patients (95 %) with unknown histology had at least 1 recurrence whereas in the main group only 63 % had recurrence. The 2 differences support a conclusion that these 22 patients form a group with poor prognosis.

It is shown in the clinical section (Jones *et al.*, 1968) that females have a higher, though not significantly higher, 5 year survival rate. In this group of 22 patients, the percentage of females is higher than that in the main group and this proportion in favour of females might be expected to improve the survival figures. If the sex ratio had been similar the 5 year survival rate would probably have been even lower.

The conclusion from the examination of these 2 sets of data is that the group for whom no histology was available is one with poor prospect of survival and is dissimilar from the main group on which tabulations and tests have been carried out.

A justification for basing the analysis on 89 cases with known histology is that differences will be understated and conclusions reached from significant values are unlikely to be wrong. Where borderline significance is shown, it should be borne in mind that the inclusion of further cases might have given a larger difference and a possible significant result.

### RESULTS

### *Presence of Histological Features*

The presence of 2 features was shown to have an effect upon 5 year survival (Table I).

TABLE I.—*Five Year Survival Rates According to:* (1) *Deep or Superficial Spread.* (2) *Grade of Mitosis.*

| Histology | Grade | Dead | Alive No. | Alive % | | Total |
|---|---|---|---|---|---|---|
| Depth of spread (sweat glands) | Deep | 31 | 2 | ( 6) | $S_1$ | 33 |
| | Superficial | 24 | 31 | (56) | | 55 |
| | Not assessed | 1 | — | — | | 1 |
| Mitosis | 2–3 | 28 | 9 | (24) | $S_2$ | 37 |
| | 0–1 | 28 | 24 | (46) | | 52 |

$S_1$ = Significant at 0·01 probability level.
$S_2$ = Borderline significance at 0·05 probability level.

(1) The difference in the survival rate when deep spread was present was highly significant, indicating that the presence of this feature militates strongly against survival.

(2) The difference in the survival rate when a high grade of mitosis was present reached borderline significance at the 0·05 probability level. Thus the presence of a high grade of mitosis reduces the time of survival.

Table I shows that out of 33 patients with lesions deep to sweat gland, only 2 (6 %) survived for 5 or more years, whereas out of 55 patients with superficial lesions, 31 (56 %) survived. The difference is highly significant ($\chi^2 = 20{\cdot}2$, $P < 0{\cdot}01$).

The number of mitoses is similarly compared. Out of 37 patients graded 2–3, 9 (24 %) survived for 5 years; out of 52 graded 0–1, 24 (46 %) survived. This difference reaches borderline significance ($\chi^2 = 3{\cdot}53$, $P = 0{\cdot}06$).

No other histological feature examined separately was shown to affect the survival rates to a significant degree. The series does show, however, that a consistently lower rate of survival is experienced when such features are present or present in a marked state (Table II). The consistency of the lower rates—shown for all but 1 of 10 characteristics—permits the inference that these features have *some* effect upon survival. The 1 characteristic which is dissimilar—presence of spindle cells—is analysed more fully in the following sections.

Table II shows the number and percentage of survivors when each of the histological features examined was present or absent. All differences in this table can be ascribed to chance but it is apparent that survival rates are consistently poorer when the feature is present. When considering this table, it should

TABLE II.—*Presence or Absence (or Grade) of Histological Features Compared by 5 Year Survival Rate*

| Histology | Grade | Dead | Alive No. | Alive % | Total |
|---|---|---|---|---|---|
| Vessel invasion | Present | 6 | 1 | (14) | 7 |
| | Absent | 50 | 32 | (39) | 82 |
| Pigmentation | 2–3 | 26 | 13 | (33) | 39 |
| | 0–1 | 30 | 20 | (40) | 50 |
| Epidermal hyperplasia | 2–3 | 18 | 7 | (28) | 25 |
| | 0–1 | 37 | 26 | (41) | 63 |
| | Not assessed | 1 | — | — | 1 |
| Polypoid | Present | 39 | 21 | (35) | 60 |
| | Absent | 16 | 12 | (43) | 28 |
| | Not assessed | 1 | — | — | 1 |
| Ulceration | Present | 46 | 26 | (36) | 72 |
| | Absent | 9 | 7 | (44) | 16 |
| | Not assessed | 1 | — | — | 1 |
| Round cell infiltration | 2–3 | 15 | 8 | (35) | 23 |
| | 0–1 | 38 | 25 | (40) | 63 |
| | Not assessed | 3 | — | — | 3 |
| Naevus pattern | 2–3 | 32 | 18 | (36) | 50 |
| | 0–1 | 21 | 14 | (40) | 35 |
| | Not assessed | 3 | 1 | — | 4 |
| Giant cells | 2–3 | 12 | 5 | (29) | 17 |
| | 0–1 | 42 | 28 | (40) | 70 |
| | Not assessed | 2 | — | — | 2 |
| Epithelial cell | 2–3 | 46 | 26 | (36) | 72 |
| | 0–1 | 10 | 7 | (41) | 17 |
| Spindle cell | 2–3 | 13 | 10 | (43) | 23 |
| | 0–1 | 43 | 23 | (35) | 66 |

be borne in mind that features have been examined independently and the presence of 1 feature does not necessarily mean that others are absent. The non-significant differences in this table did not warrant further breakdown but a more detailed analysis has been made of the one feature which gave a contrary result, namely, spindle cells.

### *Depth of Spread and Mitosis*

Superficial spread and low grade mitosis (<1 mitosis/5 H.P.F.) have been shown to give a relatively good rate of survival when considered separately

TABLE III.—*Groups A and B Compared by 5 Year Survival Rate*

*Group A*. Superficial spread and < 1 mitosis/5 H.P.F.
*Group B*. Deep spread or > 1 mitosis/5 H.P.F. or both

| Group | Dead | Alive No. | Alive % | | Total |
|---|---|---|---|---|---|
| A | 14 | 23 | (62) | S | 37 |
| B | 42 | 10 | (20) | | 52 |
| | 56 | 33 | — | | 89 |

S = Significant at 0·01 probability level.

(Table I). When both characteristics are present Table III, Group A, the survival rate is further improved—62 % of such patients surviving for 5 years. The difference in the survival rates between Group A and all other patients in the investigation who had either deep spread or high grade mitosis or both (Table III, Group B) was statistically significant ($\chi^2 = 15{\cdot}3$, $P < 0{\cdot}01$).

Groups A and B were then compared by histological features but it was apparent that depth of spread and mitotic rate were overriding influences on survival. Only two features—polypoid lesions and epidermal hyperplasia—departed from the pattern so far found (Table IV).

TABLE IV.—*Survival of Patients in Groups A and B* when:*

(a) Polypoid lesions were present/absent.
(b) Epidermal hyperplasia was present in a marked/minimal degree.

| | | Group A (37) | | | Group B (52) | | |
|---|---|---|---|---|---|---|---|
| | | | Survivors at 5 yrs. | | | Survivors at 5 yrs. | |
| | | No. | No. | % | No. | No. | % |
| Polypoid lesions | Present | 23 | 12 | (52) | 37 | 9 | (24) |
| | Absent | 14 | 11 | (79) | 14 | 1 | ( 7) |
| | Not assessed | | | | 1 | — | |
| Epidermal hyperplasia | Grades 2–3 | 13 | 7 | (54) | 12 | Nil | |
| | Grades 0–1 | 24 | 16 | (67) | 39 | 10 | (26) |
| | Not assessed | | | | 1 | — | |

* Defined in Table III.

*In Group A* the relatively lower survival rate which has already been shown to obtain when a feature is present, is also shown here.

*In Group B* there was an anomalous finding that apparently the absence of a polypoid lesion gave a worse prognosis (7 %) than did the presence of such a lesion (24 %). Unfortunately it was not possible to test this finding statistically as there were only 14 cases in Group B without a polypoid lesion and of this number we would have expected 3 persons to survive 5 years. In the issue only 1 survived and so it is clear that no real weight can be attached to this provocative finding.

No patient survived when epidermal hyperplasia was present in a marked degree. Thus in this series when deep spread, a high mitotic rate and epidermal hyperplasia were present, no patient survived for 5 years.

The finding in the clinical section (Jones *et al.*, 1968) that males over 55 years of age form a poor survival group is inherent in the results shown from the comparison of Groups A and B. Group A comprised 45 % males over 55 years of age and Group B, 57 %. It is a reasonable conclusion that the course of the disease in older males tends to take a more severe form.

Further analysis was made of depth of spread and mitosis and their relationship to other histological features.

### *DEPTH OF SPREAD*

*Deep melanoma* (33 patients)

Of 33 patients who had deep spread, only 2 survived for 5 years.

*Superficial melanoma* (55 patients)

The 5-year survival rates are given below:

| Feature | | Present (or marked degree) | | Absent (or minor degree) |
|---|---|---|---|---|
| Polypoid | . | 51 % (37) | . | 67 % (18) |
| Ulceration | . | 55 % (44) | . | 64 % (11) |
| Epidermal hyperplasia | . | 41 % (17) | . | 63 % (38) |
| Pigmentation | . | 55 % (22) | . | 58 % (33) |
| Round cell infiltration | . | 50 % (16) | . | 59 % (39) |
| Naevus pattern | . | 53 % (32) | . | 62 % (21) |
| Giant cell tumour | . | 30 % (10) | . | 62 % (45) |
| Epithelial cell | . | 55 % (44) | . | 64 % (11) |
| Spindle cell | . | 71 % (14) | . | 51 % (41) |

Figures in brackets are the numbers in the group

The pattern of lower rates of survival when other histological features are present is still shown. No difference is statistically significant and only consistency of lower rates leads to a belief that this is so. The presence of spindle cells is the one feature showing an opposite effect. Seventy-one per cent of patients graded 2–3 survived for 5 years compared with 51 % grade 0–1, suggesting that in superficial melanoma the presence of spindle cells is a benign influence. In only 1 case was vessel invasion found and this patient did not survive, while 57 % (31 out of 54) of those without invasion survived.

*MITOSIS*

*Grades* 0–1 ($<1$ *mitosis*/5 *H.P.F.*) (52 patients)

The survival rates according to the presence or absence of other features are not reproduced. Small differences were shown and none reached significance level. The largest was shown when polypoid was present, 13 out of 34 (38 %) survived as against 11 out of 17 (65 %) when it was absent.

*Grades* 2–3 ($>1$ *mitosis*/5 *H.P.F.*) (37 patients)

The 5 year survival rates are given below:

| Feature | | Present (or marked degree) | | Absent (or minor degree) |
|---|---|---|---|---|
| Polypoid | . | 31 % (26) | . | 9 % (11) |
| Ulceration | . | 26 % (34) | . | Nil ( 3) |
| Epidermal hyperplasia | . | Nil ( 9) | . | 9 % (28) |
| Pigmentation | . | 23 % (13) | . | 25 % (24) |
| Round cell infiltration | . | 10 % (10) | . | 31 % (26) |
| Vessel invasion | . | Nil ( 2) | . | 26 % (35) |
| Giant cell tumour | . | 25 % ( 8) | . | 24 % (29) |
| Epithelial cell | . | 22 % (32) | . | 40 % ( 5) |
| Naevus pattern | . | 22 % (23) | . | 31 % (13) |
| Spindle cell | . | 56 % ( 9) | . | 14 % (28) S |

Figures in brackets are the numbers in the group

The rate of 5 year survival when a high proportion of spindle cells was present was significantly greater than when these cells were absent or present only in a minor degree ($\chi^2 = 4{\cdot}26, P = 0{\cdot}04$). This investigation indicates that the presence of spindle cells is associated with a higher survival rate amongst patients with a high grade of mitosis. The possibility of other influences playing a part in this relationship has not been examined.

The presence of polypoid again suggests that this feature slows down the death rate but the difference is not significant and no conclusion can be reached from these data.

### DISCUSSION

Two of the histological features considered are associated with a significant influence on the 5 year survival, depth of spread and number of mitoses. The presence, particularly in excess, of the other features is associated with poor prognosis but with the number of cases involved in this study, no other of these differences was large enough to reach formal statistical significance. Exceptionally, spindle cells were associated with improved survival time.

Depth of spread has previously been shown to influence survival in that deep lesions are associated with a shorter survival time. But various authors have used different criteria of depth of invasion. Allen and Spitz (1953) showed that of superficial melanomata, i.e. lesions with minimal dermal infiltration, 74 % survived at least 5 years compared to their overall survival rate of 26 %. Mehnert and Heard (1965) and Lund and Ihnen (1955) obtained comparable results using similar criteria—superficial (superficial to deepest extension of rate pegs) 77 %, intradermal (superficial to sweat glands) 38 % and subcutaneous 8 %, 5 year survival times. Wright's (1949) results showed that depth of invasion was of less value than degree of cellularity and cellular anaplasia. Our choice of sweat glands to demarcate superficial from deep lesions provided a statistically significant difference in 5 years survival, 56 % and 6 % respectively. We thus confirm previous reports and show the value of the use of sweat glands as a marker.

Our results show that, as in most malignant tumours, the number of mitoses are of value in prognosis. Originally we subdivided the cases into 3 groups but on analysis found that the use of 2 subgroups was adequate. In patients with lesions showing more than 1 mitosis per 5 high power fields 24 % survived 5 years and with less than 1 mitosis per 5 high power fields, 46 %. The results reached borderline significance. Allen and Spitz (1953) in their analysis of superficial lesions showed that mitoses were absent or very scanty, per section, in 72 % of 5 year survivors as compared to 53 % of those that died. Hall *et al.* (1952) using Broders' tumour classification, which includes mitosis (Grade 1 few, Grade 4 many) showed a decreased survival time in Grade 4 as compared to Grade I. We consider our finding of importance as many previous authors have denied that the number of mitoses influences prognosis (Wright, 1949; Lane *et al.*, 1958; Mehnert and Heard, 1965). We agree with Allen and Spitz (1953) that in malignant melanoma mitoses tend to be scanty.

As the depth of spread and number of mitoses were the only 2 features of marked prognostic value a further analysis was performed combining the 2 according to severity. We have shown that cases with superficial melanoma and less than 1 mitosis per 5 high power fields show a significantly better 5 year survival, 62 %

compared to 20 % for all others with either or both deep spread and more than 1 mitosis per 5 high power fields. These 2 histological features show an overriding influence on survival when compared to other features. In lesions with high grade mitosis and deep spread the presence of polypoid feature slightly increased survival in this series, while the presence of marked epidermal hyperplasia decreased the 5 year survival to nil. Allen and Spitz (1953) showed that epidermal hyperplasia is usually associated with deep spread and a poor prognosis but Mehnert and Heard (1965) failed to confirm this association.

Further analysis of the relationship of superficial spread alone to other histological features yielded no significant results but the presence of any other feature, with one exception, tends to be associated with decreased 5 year survival. The exception, spindle cells, when present, tends to be associated with an improved survival rate. A similar analysis of the relationship between deep spread alone and other features was impossible as only 2 cases survived 5 years.

The relationship between high mitotic count alone and other features showed only one significant association. When associated with spindle cells the survival rate was significantly improved.

We have thus shown that the presence of spindle cells particularly in excess (66 %) in superficial and high mitotic rate tumours is associated with improved survival. Wright (1949) also showed that spindle cells are a benign influence and described a separate spindle cell variety of malignant melanoma with a better than average prognosis. Callender (1931) showed a definite benign influence of spindle cells in ocular melanoma but other authors have failed to show this association in skin melanomas (Allen and Spitz, 1953; Mehnert and Heard, 1965).

In common with previous authors we failed to show that the presence or grade of the following histological features definitely influence prognosis: naevus pattern, giant cell, pigmentation, lymphocytic infiltration, vessel invasion.

The lack of influence and possible deleterious effect of lymphocytic infiltration on prognosis is noteworthy. This has been shown to be associated with improved survival rates in carcinoma of breast (Black, 1965) and possibly reflects a host *versus* tumour cell mediated immune response.

Very few of our cases showed vessel invasion, 7 out of 89 (8 %) and only 1 of the 7 survived 5 years whereas we would have " expected " 3 to do so if this finding had no effect on prognosis. Clearly we cannot comment on the relationship between vessel invasion and prognosis with such small numbers. Mehnert and Heard (1965) found that 14 % of their cases had vessel invasion and with this number they were able to demonstrate a worsening of the survival rate which was statistically significant.

### SUMMARY

A detailed histological study of 89 malignant melanomas with an assessment of the relationship of 14 histological features to 5 year survival is reported.

(1) Depth of spread (related to sweat glands) shows a significant relationship. Deep spread is associated with poor survival.

(2) Mitotic count is of borderline significance. High mitotic count, $>1$ mitosis/5 high power fields, signifies a poor prognosis.

(3) 1 and 2 show an over-riding influence on survival compared to all other features. It was nevertheless noted in this series that:

(a) Polypoid shape in deep, high mitotic grade tumours is associated with slightly improved survival.

(b) Well marked epidermal hyperplasia in deep, high mitotic grade tumours decreased survival to nil.

(4) A preponderence of spindle malignant cells in high mitotic grade lesions is significantly associated with improved survival. In superficial lesions such an association is marginal.

(5) The presence and degree of pigmentation, ulceration, round cell infiltration, naevoid pattern, giant cells, epithelial malignant cells and vessel invasion tend to be associated with a poorer prognosis.

(6) Junctional activity and absence of hair were always found and are not analysed as they are considered to be essential diagnostic criteria of malignant melanomas.

Our thanks are due to many surgical, radiotherapy and pathology colleagues for access to records and materials, and the Tenovus Fund for Cancer Research for financial assistance to one of us (W.M.J.).

### REFERENCES

Allen, A. C. and Spitz, S.—(1953) *Cancer, N.Y.*, **6**, 1.

Black, M. M.—(1965) *Prog. Cancer.*, **1**, 26.

Bloodgood, J. C.—(1922) *J. Am. med. Ass.*, **79**, 576.

Callender, G. R.—(1931) *Trans. Am. Acad. Ophthal. Oto-lar.*, **36**, 131.

Hall, J. R., Phillips, C. and White, R. R.—(1952) *Surgery Gynec. Obstet.*, **95**, 184.

Jones, W. M., Williams, W. J., Roberts, M. M. and Davies, K.—(1968) *Br. J. Cancer*, **22**, 437.

Lane, N., Lattes, R. and Malm, J.—(1958) *Cancer, N.Y.*, **11**, 1025.

Lund, R. H. and Ihnen, M.—(1955) *Surgery, St Louis*, **38**, 652.

Mehnert, J. H. and Heard, J. E.—(1965) *Am. J. Surg.*, **110**, 168.

Wright, C. J. E.—(1949) *J. Path. Bact.*, **61**, 507.